# Association of Body Mass Index with Hemoglobin Concentration and Iron Parameters in Iranian Population

**DOI:** 10.1155/2014/525312

**Published:** 2014-02-10

**Authors:** Akram Ghadiri-Anari, Narjes Nazemian, Hassan-Ali Vahedian-Ardakani

**Affiliations:** ^1^Yazd Diabetes Research Center, Shahid Sadoughi University of Medical Science, P.O. Box 8916657343, Yazd, Iran; ^2^Department of Internal Medicine, Shahid Sadoughi University of Medical Sciences and Health Services, Yazd, Iran

## Abstract

*Background*. Studies have reported that obesity has an adverse effect on iron metabolism. Obesity is characterized by chronic, low-grade, systemic inflammation and anemia of chronic disease with elevated serum ferritin and decreased level of serum iron, transferrin saturation, and hemoglobin. Therefore, we examined the association of body mass index with hemoglobin concentration and iron parameters in this study. *Methods*. This cross-sectional study was conducted in Yazd to assess the relation of body mass index with hemoglobin and iron parameters among 406 adult patients 18–65 years old. Diabetes and conditions that could influence body iron stores were excluded. *Results*. There is no difference in hemoglobin concentrations, MCV, serum iron, TIBC, transferrin saturation index, and ferritin between normal weight, overweight, and obese persons. *Conclusion*. Nutritional status of persons and intake of high iron foods by obese persons should be considered. Also, other inflammatory markers should be evaluated in the future studies.

## 1. Introduction 

The increasing prevalence of obesity is a global health concern. In 2005, an estimated 400 million adults worldwide were obese [1].

Obesity increases the risk of type 2 diabetes, hypertension, heart disease, stroke, dyslipidemia, osteoarthritis, gynecological problems, sleep apnea, and respiratory problems. In addition, studies have reported that obesity has an adverse effect on iron status [2–6]. Studies on obese mice showed high level of hepatic hepcidin mRNA expression and suggested that lower hepatic iron status in obese animals might be associated with inflammation. Hepcidin is a homeostatic regulator of iron metabolism that restricts intestinal iron absorption and is known as a mediator of inflammation [7]. Rapid dietary and lifestyle changes in Mexico have produced a double burden of malnutrition with under- and over-nutrition occurring in the same population [8].

Obesity is characterized by chronic, low-grade, systemic inflammation, which, in turn, has been associated with anemia of chronic disease specifically, elevated serum ferritin and low serum iron, transferrin saturation (TS), and hemoglobin. Studies have shown that the CRP concentration decreases significantly after massive weight reduction. This decrease indicates that fat mass plays an important role in the production of CRP. CRP is the main acute phase protein and it is also a sensitive marker of systemic inflammation [9]. A large study confirmed that overweight children were twice as likely to be iron deficient as normal weight children [10]. Ausk and coworker showed that increasing BMI is associated with higher serum ferritin levels and lower serum levels of iron and transferrin saturation. However, hemoglobin concentration was not lower in overweight and obese persons compared with normal-weight persons [11].

Another study conducted among 740 school children in Iran showed that prevalence of iron deficiency increased with the subject's body mass index [12].

Therefore, we examined the association of body mass index with hemoglobin concentration and iron parameters in adult populations in Yazd province of Iran. We hypothesized that overweight and obese adults have lower iron status than their normal weight counterparts do and that this would be associated with obesity-related inflammation.

## 2. Materials and Methods

This was a descriptive cross-sectional study conducted among 406 adult patients (18–65 years old) who came to the obesity clinic of yazd diabetes research center in Yazd province (Iran) from January 2011 to January 2013.

The exclusion criteria for this study were conditions that could influence body iron store such as clinical evidence of hemorrhage in the preceding six months, iron or folate or vitamin B12 treatment in the previous year, blood donation within the previous six months, Concomitant Infections, chronic diseases, and diabetes mellitus. Also, persons who were on specific regimen were excluded.

Anthropometric measurements, including height which was measured without shoes against a wall-fixed tape and weight which was measured with light clothing and without shoes on a platform scale with a 1.0 kg subtraction to correct for the weight of the clothing, were taken.

The body mass index (BMI) was calculated as weight/height^2^ (kg/m^2^). For adults, overweight and overall obesity were defined as 25 ≤ BMI < 30 and BMI ≥ 30, respectively, according to the latest World Health Organization (WHO) criteria [13].

Informed written consent was obtained from all participants, and the study was approved by the hospital's human ethics committee. All laboratory measurements were performed on fasting blood samples. Ferritin was measured by a turbidimetric fixed rate method. Serum iron was measured by a photometric color test for clinical chemistry analyzers. transferrin was measured by a turbidimetric end-point method. transferrin saturation index was calculated as serum iron divided by transferrin. Comparisons between groups were performed using analysis of variance and Student's *t*-tests for continuous variables and Chi-square test for categorical variables. The relationships between the continuous variables were examined by Pearson's linear correlation test. Significance was accepted at the level of *P* ≤ 0.05. All statistical procedures were performed by using SPSS 16.0 for Windows (SPSS Inc., Chicago, IL).

## 3. Results

In this cross-sectional study, we collected data from 406 persons (18–65 years old) that were referred to the obesity clinic of Yazd diabetes research center in Yazd province in Iran from January 2012 to January 2013. In this study, were %22.1 male (90 patients) and were %77.9 female (316 patients).

Demographic characteristics, anthropometric measurements, and biochemical indexes by BMI categories are shown in Table 1. In this study, 32.2% of total was normal weight and 37.7% and 30.1% were overweight and obese, respectively. No difference in hemoglobin concentrations, MCV, serum iron, TIBC, transferrin saturation index, and ferritin was found between all BMI groups (Table 1).

## 4. Discussion

Obesity is a risk factor for many diseases such as type 2 diabetes, hypertension, heart disease, stroke, dyslipidemia, osteoarthritis, gynecological problems, sleep apnea, and respiratory problems. Also, studies showed that obesity has an adverse effect on iron status [2–6].

The results of our study showed that BMI had no correlation with hemoglobin, MCV, serum iron, TIBC, transferrin saturation index, and ferritin. This result is against the previous results that were mentioned [9–12].

Obesity was diagnosed in 16.7% of children in the study by Sharma and coworkers. Children with high BMI group had significantly lower serum iron levels and markedly higher CRP and soluble transferrin receptor levels. Serum iron exhibited a significant correlation with both the BMI and CRP. Ferritin concentration was similar in the group of obese as well as normal weight children [14]. Lower levels of serum iron and transferrin saturation have been observed in obese adults compared to nonobese matched controls, with fat mass shown to be a significant negative predictor of serum iron concentration [15].

The cause of the iron deficiency of obesity is unclear. Iron deficiency in obese individuals may be a result of low iron intake (due to an unbalanced diet), reduced iron absorption in the small intestine, and greater iron requirements caused by a larger blood volume. In addition, obesity is associated with a chronic low grade inflammation state. For this reason, sequestration of iron through an inflammatory mediated mechanism can be one of the proposed causes of iron deficiency in obesity [15, 16].

We exclude diabetic patients in our study. Lecube and coworker showed that, in postmenopausal women with obesity, serum ferritin levels were significantly higher in obese women with metabolic syndrome in comparison with obese women without metabolic syndrome. There is no difference in the other markers of iron status such as iron, soluble transferrin receptor, transferrin, and transferrin saturation index. Diabetic patients had higher ferritin levels than nondiabetic patients. Nondiabetic patients with metabolic syndrome also showed higher ferritin levels than nondiabetic patients without metabolic syndrome. Among the components of metabolic syndrome, only diabetes was independently associated with serum ferritin levels in both the whole group and in patients with metabolic syndrome [17]. These findings may be explained in our results (exclusion of diabetic patients). It seems that diabetes had a powerful predictor for inflammation and anemia of chronic disease rather than for obesity itself.

Another cause of our finding (normal markers of iron status in obese persons) may be well nutritional status of obese people, for example, intake of high iron foods.

The main limitation of our study is the use of limited laboratory indexes such as iron, transferrin, and ferritin which were selected because they are readily available in many biochemical tests. Other inflammatory parameters such as CRP, interleukin-6 (IL-6), and tumor necrosis factor alpha (TNF-alpha) should be studied as well in the future. Also studies with greater sample size are recommended.

## 5. Conclusions

BMI had no correlation with hemoglobin, MCV, serum iron, TIBC, transferrin saturation index, and ferritin. Nutritional status of persons and intake of high iron foods by obese persons should be considered. Also other inflammatory markers should be evaluated in the future studies.

## Figures and Tables

**Table 1:** Baseline characteristics of patients according to BMI.

Variable	Normal weight (*n* = 131; 32.2%)	Overweight (*n* = 153; 37.7%)	Obese (*n* = 122; 30.1%)	Total (406, 100%)	*P* value
Age (y)	34 ± 13	42.9 ± 13.4	43.9 ± 12	40.6 ± 13.9	0.001
BMI (kg/m^2^)	21.76 ± 2.8	27.8 ± 1.4	35.3 ± 5.9	28.1 ± 6.5	0.001
Hemoglobin (mg/dL)	13.2 ± 1.4	13.7 ± 6.3	13.5 ± 1.2	13.5 ± 4	0.58
MCV	84.6 ± 8.4	83.3 ± 8.5	84.3 ± 7.9	84.1 ± 8.3	0.38
Serum iron (*μ*g/dL)	81.9 ± 32.3	76 ± 27.7	79.8 ± 29	79.1 ± 29.7	0.27
TIBC (*μ*g/dL)	327 ± 51	343 ± 48	334 ± 66.2	335.6 ± 55.7	0.06
TS (%)	26.9 ± 20.4	22.7 ± 8.7	26.2 ± 20.6	25.15 ± 17.1	0.08
Ferritin (ng/mL)	88 ± 40	76.8 ± 43	82.2 ± 53	82.1 ± 51	0.53

## References

[1] World Health Organization (2006). *Obesity and Overweight*.

[2] Tussing-Humphreys LM, Liang H, Nemeth E, Freels S, Braunschweig CA (2009). Excess adiposity, inflammation, and iron-deficiency in female adolescents. *Journal of the American Dietetic Association*.

[3] Pinhas-Hamiel O, Newfield RS, Koren I, Agmon A, Lilos P, Phillip M (2003). Greater prevalence of iron deficiency in overweight and obese children and adolescents. *International Journal of Obesity*.

[4] Nead KG, Halterman JS, Kaczorowski JM, Auinger P, Weitzman M (2004). Overweight children and adolescents: a risk group for iron deficiency. *Pediatrics*.

[5] Yanoff LB, Menzie CM, Denkinger B (2007). Inflammation and iron deficiency in the hypoferremia of obesity. *International Journal of Obesity*.

[6] Lecube A, Carrera A, Losada E, Hernández C, Simó R, Mesa J (2006). Iron deficiency in obese postmenopausal women. *Obesity*.

[7] Chung J, Kim MS, Han SN (2011). Diet-induced obesity leads to decreased hepatic iron storage in mice. *Nutrition Research*.

[8] Food and Agriculture Organization of the United Nations (2006). The double burden of malnutrition, case studies from six developing countries. *FAO Food and Nutrition Paper*.

[9] Chen S-B, Lee Y-C, Ser K-H (2009). Serum C-reactive protein and white blood cell count in morbidly obese surgical patients. *Obesity Surgery*.

[10] Nead KG, Halterman JS, Kaczorowski JM, Auinger P, Weitzman M (2004). Overweight children and adolescents: a risk group for iron deficiency. *Pediatrics*.

[11] Ausk KJ, Ioannou GN (2008). Is obesity associated with anemia of chronic disease? A population-based study. *Obesity*.

[12] Moayeri H, Bidad K, Zadhoush S, Gholami N, Anari S (2006). Increasing prevalence of iron deficiency in overweight and obese children and adolescents (Tehran Adolescent Obesity Study). *European Journal of Pediatrics*.

[13] WHO (2000). Obesity: preventing and managing the global epidemic. Report of a WHO consultation. *World Health Organization Technical Report Series*.

[14] Sharma AP, McKenna AM, Lepage N, Nieuwenhuys E, Filler G (2009). Relationships among serum iron, inflammation, and body mass index in children. *Advances in Pediatrics*.

[15] Menzie CM, Yanoff LB, Denkinger BI (2008). Obesity-related hypoferremia is not explained by differences in reported intake of heme and nonheme iron or intake of dietary factors that can affect iron absorption. *Journal of the American Dietetic Association*.

[16] Yanoff LB, Menzie CM, Denkinger B (2007). Inflammation and iron deficiency in the hypoferremia of obesity. *International Journal of Obesity*.

[17] Lecube A, Hernández C, Pelegrí D, Simó R (2008). Factors accounting for high ferritin levels in obesity. *International Journal of Obesity*.

